# Local Differential Privacy in the Medical Domain to Protect Sensitive Information: Algorithm Development and Real-World Validation

**DOI:** 10.2196/26914

**Published:** 2021-11-08

**Authors:** MinDong Sung, Dongchul Cha, Yu Rang Park

**Affiliations:** 1 Department of Biomedical Systems Informatics Yonsei University College of Medicine Seoul Republic of Korea; 2 Department of Otorhinolaryngology Yonsei University College of Medicine Seoul Republic of Korea

**Keywords:** privacy-preserving, differential privacy, medical informatics, medical data, privacy, electronic health record, algorithm, development, validation, big data, medical data, feasibility, machine learning, synthetic data

## Abstract

**Background:**

Privacy is of increasing interest in the present big data era, particularly the privacy of medical data. Specifically, differential privacy has emerged as the standard method for preservation of privacy during data analysis and publishing.

**Objective:**

Using machine learning techniques, we applied differential privacy to medical data with diverse parameters and checked the feasibility of our algorithms with synthetic data as well as the balance between data privacy and utility.

**Methods:**

All data were normalized to a range between –1 and 1, and the bounded Laplacian method was applied to prevent the generation of out-of-bound values after applying the differential privacy algorithm. To preserve the cardinality of the categorical variables, we performed postprocessing via discretization. The algorithm was evaluated using both synthetic and real-world data (from the eICU Collaborative Research Database). We evaluated the difference between the original data and the perturbated data using misclassification rates and the mean squared error for categorical data and continuous data, respectively. Further, we compared the performance of classification models that predict in-hospital mortality using real-world data.

**Results:**

The misclassification rate of categorical variables ranged between 0.49 and 0.85 when the value of ε was 0.1, and it converged to 0 as ε increased. When ε was between 10^2^ and 10^3^, the misclassification rate rapidly dropped to 0. Similarly, the mean squared error of the continuous variables decreased as ε increased. The performance of the model developed from perturbed data converged to that of the model developed from original data as ε increased. In particular, the accuracy of a random forest model developed from the original data was 0.801, and this value ranged from 0.757 to 0.81 when ε was 10^-1^ and 10^4^, respectively.

**Conclusions:**

We applied local differential privacy to medical domain data, which are diverse and high dimensional. Higher noise may offer enhanced privacy, but it simultaneously hinders utility. We should choose an appropriate degree of noise for data perturbation to balance privacy and utility depending on specific situations.

## Introduction

Big data is a core factor in the renovation of medicine. The raw data have low utility; however, applying algorithms such as machine learning (ML) enables us to make the most of these data [1]. Unlike rule-based systems, ML algorithms are data driven and require a large amount of data. Particularly, conventional ML approaches require centralized data for learning. To obtain this substantial amount of data, it is necessary to exchange data among different organizations to develop an effective ML model.

However, the exchange of data between different parties causes privacy problems, and there are increasing concerns about privacy violations by large companies [2]. Medical data that mostly contain sensitive information should be appropriately protected when shared with third parties. The European Union’s General Data Protection Regulation [3] and the United States’ Health Insurance Portability and Accountability Act of 1996 (HIPAA) [4] recognize this problem and require users’ privacy to be strengthened. Medical data have various distinct properties in addition to their sensitive attributes. For example, serum glucose levels are continuous, whereas medical histories are usually recorded using categorical values. Medical data also contain multimodal values: some of the data may be obtained from blood tests, whereas others may originate from radiologic and physical examination tests.

Deidentification is defined as “the removal or replacement of personal identifiers so that it would be difficult to reestablish a link between the individual and his or her data [5].” Especially, in the HIPAA, data is considered as deidentified when specified data elements are removed [4]. Anonymization is defined as “the irreversible removal of the link between the individual and his or her medical record data to the degree that it would be virtually impossible to reestablish the link [5].” In such a case, the anonymized data could never be reidentified using the data in the underlying data sets. There are three primary ways to anonymize these data: suppression, generalization, and noise addition [6]. Deidentification may not necessarily be anonymized. That is, anonymization is a subset of deidentification. Following anonymization, three main measures to identify the privacy risk can be evaluated: *k*-anonymity [7], *l*-diversity [8], and *t*-closeness [9]. Deidentification tools, such as ARX [10], offer seamless privacy protection through feature generalization and the suppression of records.

Differential privacy [11], which entails a semantic model, is another data privacy approach. Compared to syntactic anonymity, it requires less domain knowledge and is inherently robust to linkage attacks combined with domain knowledge. Moreover, differential privacy is considered to be a de facto standard for private data analysis or publishing [12,13]. Technology companies such as Apple and Google have attempted to apply differential privacy to protect the privacy of mobile data [14,15]. Moreover, the rapid development of the Internet of Things (IoT) should consider privacy risk [16]. Researchers have been actively applying differential privacy to the IoT, such as automatically driving cars [17] and sensors [16]. In ML, personal information can be leaked. Applying differential privacy to the deep learning model can overcome this threat [18,19], and the health care domain is no exception. Several studies have been performed in the health care domain. For example, Kim et al [20] introduced a local differential privacy algorithm for health data streams. Also, Suriyakumar et al [21] investigated the feasibility of differentially private stochastic gradient descent in a health care setting with the influential function. Most studies focus on a data set that has only a few features and focus on differential privacy in the deep learning model.

In this study, we focused on local differential privacy with regard to multivariate medical data. We applied differential privacy with diverse parameters and checked (1) the feasibility of training our algorithms with synthetic data and (2) the balance between data privacy and utility with regard to ML techniques.

## Methods

**Figure 1:**
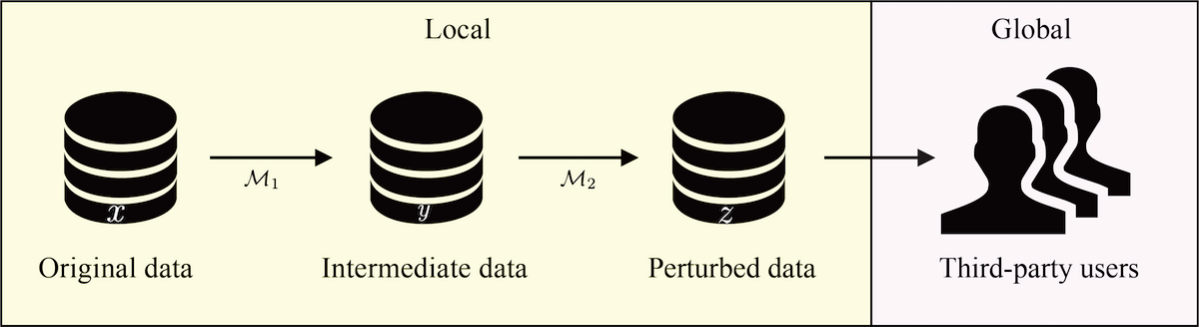
Differential privacy upon data request from third party users. The owner perturbs the original data to preserve privacy before sending the data externally. The third-party user can be either a curator or the final user. <inline-graphic xlink:href="medinform_v9i11e26914_fig5.png" xlink:type="simple" mimetype="image"/>: bounded Laplacian method; <inline-graphic xlink:href="medinform_v9i11e26914_fig6.png" xlink:type="simple" mimetype="image"/>: discretization postprocessing.

Figure 1 presents the workflow employed to achieve differential privacy in this study. When a user requests data, we perturb the data using the bounded Laplacian method (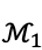) and discretization postprocessing (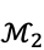) to provide high-fidelity data while preserving the privacy of the original data.

### The Value of ε for Local Differential Privacy

Dwork et al [22] defined *ε*-differential privacy as a randomized function. For adjacent data Y_1_ and Y_2_, function κ is (*ε*, *δ*)–differentially private if

P[κ(Y_1_) ∈ S] ≤ ε ∙ P[κ(Y_2_) ∈ S] + δ

where *S ⊂ Range*(κ). Local differential privacy is a specific case in which the random function or perturbation is applied by data owners, not by central aggregators.

### Bounded Laplacian Method

Before applying local differential privacy, all variables were normalized to a range between –1 and 1. First, we applied the bounded Laplacian method. Because a conventional Laplacian distribution yields an infinite boundary, it entails some limitations when applied to clinical domains. For example, respiratory rates, which are supposed to be a positive number, may become negative after applying the conventional Laplacian method, which is illogical. There are two methods to overcome this problem: the truncation method and the bound method [23]. We focused on the latter to minimize the probability of data manipulation because changes in data in the medical domain may have a considerable impact on the desired outputs.

We used the bounded Laplacian function proposed by Holohan et al [23], assuming that the input variable is within the output domain. Given *b* > 0, *W_q_*: *Ω* → *D*, for each *q* ∈ *D*, we defined the probability density function 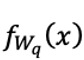 as:




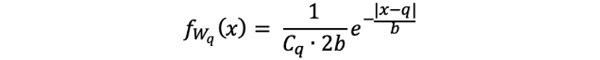




where




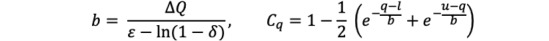




We set *δ*=0,*l* (lower bound) as –1, *u* (upper bound) as 1, and ∆*Q* as 2 in our experiments and adjusted *ε* to measure the effect of the privacy changes.

### Discretization Postprocessing for Discrete Variables

Because we applied the bounded Laplacian method to perturb the given data to a range between –1 and 1 in a continuous manner, there are infinite possibilities for a given input. Many medical domain variables are categorical (either ordinal or nominal), such as medicosurgical histories. Therefore, following the application of the bounded Laplacian method, additional postprocessing was performed for categorical variables. We distributed the intermediate output of the given data over the Bernoulli distribution, similar to the method proposed by Yang et al [17]. The perturbed data *y* ∈ [–*C*,*C*] were separated into m pieces, where m is the cardinality of the original input variable (a positive integer). We first shifted the range [–*C*,*C*] to [0, *m*] by equally dividing the space, which resulted in 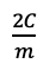 intervals. Therefore, for given perturbed data y, we obtain the following:




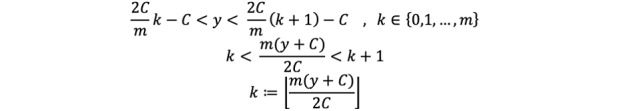




After calculating k, the Bernoulli probability *p* was sampled such that




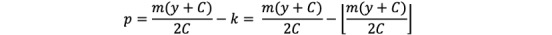




which is the distance between two adjacent possibilities. Finally, we discretized the perturbed data *y* concerning the Bernoulli probability *p* such that




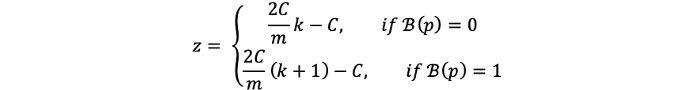




where 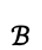 denotes the Bernoulli distribution function.

### Data Set for Validation

We used simulated (randomly generated) data for initial validation to ensure that the bounded Laplacian method functions as expected. To simulate real-world use, we used the eICU Collaborative Research Database [24]. First, to evaluate the extent to which the proposed differential privacy algorithms effectively perturbed the given original data, we used the misclassification rate for categorical variables and mean squared error (MSE) for continuous variables when measuring the similarity between two data sets. Second, to evaluate the adverse effect of differential privacy on the utility of the data set, we compared the accuracy of predicting the mortality rate following intensive care unit admission using Acute Physiology and Chronic Health Evaluation (APACHE) [25] scoring variables under various ε values. The data set contained intubated, ventilation, dialysis, medication status (cardinality: 2), eyes (cardinality: 4), motor (cardinality: 5), and verbal status (cardinality: 6) as categorical variables. Urine output, temperature, respiratory rate, sodium, heart rate, mean blood pressure, pH, hematocrit, creatinine, albumin, oxygen pressure, CO_2_ pressure, blood urea nitrogen, glucose, bilirubin, and fraction of inspired oxygen (FiO_2_) values were considered continuous variables. There were initially 148,532 patients (rows) in the data set, but after the deletion of missing values, the data set contained a total of 4740 patients (3597 who were alive and 1143 who had died). The following ML methods were used for mortality prediction: decision tree, K-nearest neighbor, support vector machine, logistic regression, naïve Bayes, and random forest. The data were divided into training and test sets in a ratio of 80:20. All predictions were averaged using a 5-fold cross-validation method, and the scikit-learn [26] library was used with the Python programming language.

## Results

### Synthetic Data for Validation of the Bounded Laplacian Function

We created an equally spaced distribution, ranging between –1 and 1, and applied the bounded Laplacian method. In contrast to the conventional Laplacian method, which has an infinite range, the bounded method entailed a range of –1 to 1.

**Figure 2:**
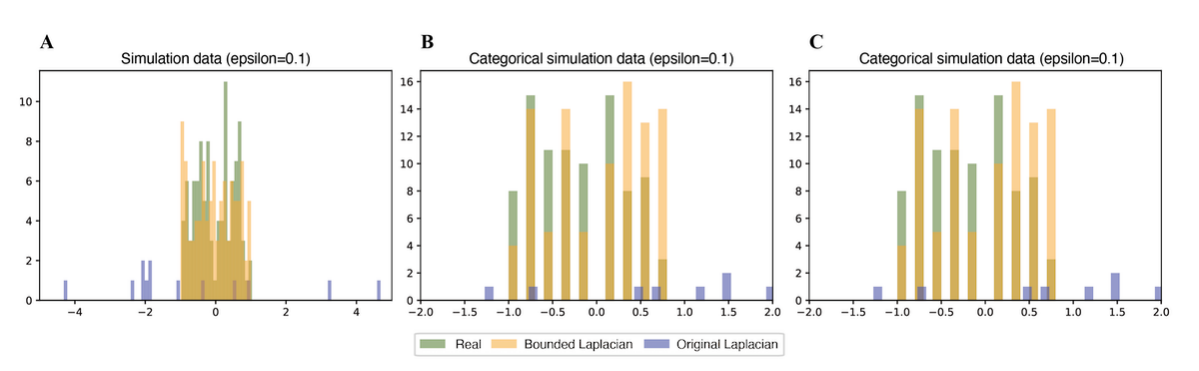
Comparison of conventional and bounded Laplacian methods using synthetic data. (A) Histogram of randomly generated continuous data ranging from –1 to 1. (B) Histogram of randomly generated categorical data, which originally ranged from 0 to 9 and were then normalized to range from –1 to 1. (C) Histogram obtained after application of discretization postprocessing to the data in (B). In all scenarios, the Laplacian method was applied with *ε*=0.1, *δ*=0.

After confirming that the bounded Laplacian method works as intended, we then created synthetic continuous data that range from –1 to 1 and applied the conventional Laplacian method and bounded Laplacian method with *ε*=0.1, *δ*=0 (Figure 2A). The original Laplacian method had out-of-range occurrences that were not present in the bounded Laplacian method. To test the categorical data and postdiscretization processing, we created a set of 100 random integers ranging from 0 to 9, then normalized them to range from –1 to 1. The original Laplacian method had some occurrences that were out of bounds. In the categorical data, the bounded Laplacian method stayed within the data range, as in the continuous data. However, some of the categorical values were not initially present in the given data (Figure 2B), which is similar to the out-of-bounds condition. Therefore, additional postprocessing discretization was performed, and the algorithm showed that the discretization technique ensures that there are no nonexistent values in the categorical data (Figure 2C).

### Validation Using Real-World Data

**Figure 3:**
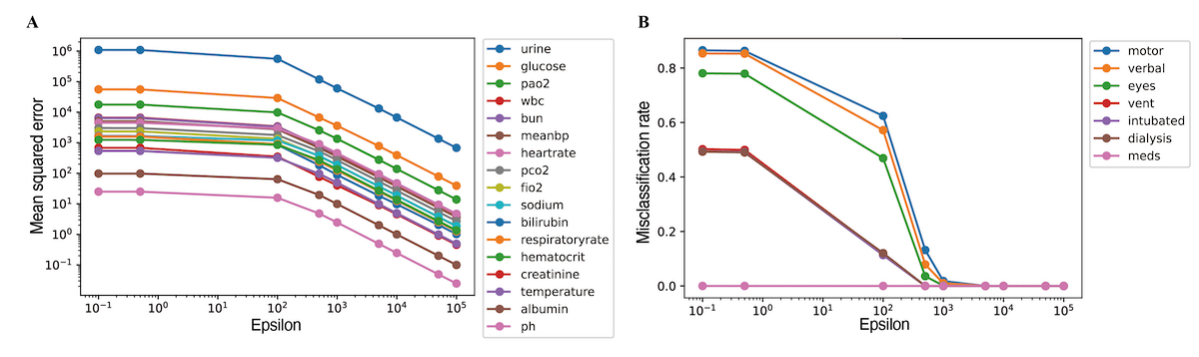
ε values and degrees of data perturbation for (A) continuous variables and (B) categorical variables. bun: blood urea nitrogen; fio2: fraction of inspired oxygen; meanbp: mean blood pressure; pao2: partial pressure of oxygen, arterial; pco2: partial pressure of carbon dioxide; wbc: white blood cells.

The eICU Collaborative Research Database [24] was used for validation. We used MSEs and misclassification rates as metrics for continuous and categorical variables, respectively, to calculate the differences between the original and perturbed data. Because of the variance between values in the original data, the MSE of continuous variables varies extensively in the case of eICU data. For example, pH and albumin are similar among different individuals, whereas heart rate and glucose have substantial differences (Figure 3A). Regarding the categorical variables, intubated, ventilation, and dialysis status are either 0 or 1, and the chance level is 0.5. The value for “eye” ranges from 1 to 4, that for “verbal” ranges from 1 to 5, and that for “motor” ranges from 1 to 6. Therefore, there were differences in the misclassification rates, especially when *ε* was small (Figure 3B). As *ε* increased, all perturbed values approached their original values for both continuous and categorical variables (Figures 3A and 3B).

**Figure 4:**
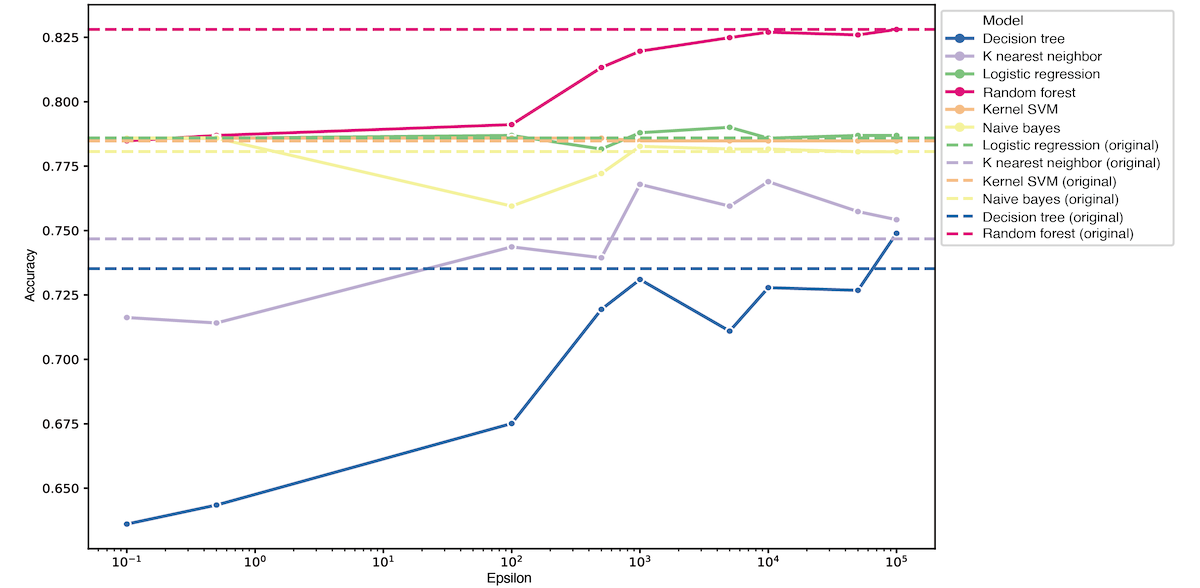
Classification accuracies among different machine learning models with respect to ε. The performance of the models developed using original data is marked with dashed lines. SVM: support vector machine.

To simulate data utility with respect to *ε*, we constructed a predictive classifier to predict mortality using the eICU data set. Note that 3,597 of the 4,740 patients (75.9%) were alive, yielding a chance level of 76%. A lower value of *ε* caused severe data perturbation, resulting in an accuracy that was near the chance level. Increasing the value of *ε* increased the performance of the classifiers, and the performance converged to the accuracy obtained using the original data (shown as dashed lines in Figure 4). This tendency was consistent among the different models, and the random forest model was the top performer.

## Discussion

### Principal Findings

In this study, we developed and validated a local differential privacy method for the medical domain. We used the bounded Laplacian method to overcome the out-of-bounds problem. In addition, we used discretization postprocessing for the categorical variables to address nonexistent categorical variables following perturbation.

Various approaches and metrics are employed when publishing microdata publicly. *k*-anonymity [7] is a metric that requires each cluster (or set of persons in medical data) to have at least *k* records so that there are at least *k* – 1 individuals that are indistinguishable. However, this metric is susceptible to reidentification through linkage attacks and applications of background knowledge. *l*-diversity was introduced to overcome these limitations; it requires each equivalent block containing sensitive information to have at least *l* appropriately represented values. This method is still vulnerable to skewness and similarity attacks [9]. *t*-closeness [9] mitigates this issue by requiring an equivalence class to have a distance of less than *t* (the earth mover distance) between the distribution of a sensitive attribute and that of the overall data. However, using the earth mover distance makes it difficult to identify the closeness between *t* and the gained knowledge. In addition, in this approach, the distribution of sensitive attributes in the equivalence class must be similar to that in the entire data set.

In contrast to these privacy metrics and methods, *ε*-differential privacy retains the structure of the data while adding noise to prevent leakage of the original data (Figure 2). There are two main differential privacy schemas: global and local. Global differential privacy requires the database owner to trust a curator that performs data perturbation before sending the data to the requested user. Our implementation, local differential privacy, assumes the worst-case scenario by considering an untrusted curator. The leakage of a medical data set may have critical consequences because such a data set may contain sensitive information, such as disease data, medical history, and insurance status. Therefore, our method minimizes the risk of data leaks by not trusting anyone outside the network.

Medical domain data are, by nature, multidimensional and multimodal. *k*-anonymity may suffer from severe utility loss if applied to high-dimensional data [27]. *ε*-differential privacy also suffered from severe utility loss under a low *ε*, which was apparent from the low classification accuracy in predicting the mortality rate (Figure 4). Despite the fact that the given data set was multidimensional and multimodal, adjusting the value of *ε* affected all variables uniformly regardless of their data type.

Differential privacy usually has stronger tradeoffs between data utility, which we mainly focused on, and privacy [28,29]. There were high variances between variables with regard to the MSEs and misclassification rates when *ε* was low (Figure 3). As *ε* increased, all variables approached their actual values, enabling better utility at the cost of privacy; this is apparent from the accuracy of prediction shown in Figure 4. When publishing synthetically perturbed data with *ε*-differential privacy, we may consider providing the *ε* value along with the data. This additional information may provide users with insights into the degree of data perturbation.

According to the results, for our data set, we may heuristically choose an *ε* value between 10^3^ and 10^4^ and apply differential privacy methods to send the perturbed data upon the user’s request. The optimal value of *ε* varies among different data sets and utility requirements, and choosing this value is beyond the scope of this study.

A limitation of this study is that we only applied our algorithms to synthetic data, and we validated the algorithms on only one data set. However, it is likely that other data sets can also be directly employed because we used a relatively small amount of prior data knowledge in our algorithm. In addition, we excluded rows that contained null values in the database. Because medical data are high-dimensional and sparse, future studies should be conducted to address null values. The distributions of data sets affect the normalization and the perturbation process. It is better to share distributions with each institute, such as the minimum and maximum values of each column. The model would be developed from perturbed data, which can be less accurate than a model based on original data. The optimal ε value, which determines the degree of perturbation, should be set to apply to the algorithm. In this study, a value of ε between 10^3^ and 10^4^ seemed heuristically appropriate; this depends on which data or model is used.

### Conclusion

We applied local differential privacy to medical domain data, which is diverse and high-dimensional. Applying bounded Laplacian noise with discretization postprocessing ensures that no out-of-bound data are present. Higher noise may offer enhanced privacy, but it simultaneously hinders utility. Thus, choosing an appropriate degree of noise for data perturbation entails a privacy-utility tradeoff, and one should choose such parameters depending on specific situations.

## Abbreviations

APACHE: Acute Physiology and Chronic Health Evaluation
FiO2: fraction of inspired oxygen
HIPAA: Health Insurance Portability and Accountability Act
IoT: Internet of Things
ML: machine learning
MSE: mean squared error
